# *RSC Advances* Outstanding Student Paper Awards 2021

**DOI:** 10.1039/d2ra90082c

**Published:** 2022-09-21

**Authors:** Dr Laura Fisher, Executive Editor for RSC Advances

## Abstract

In 2021, *RSC Advances* launched an award series to recognise the hard work of students. These awards recognise outstanding work published in the journal, for which a substantial component of the research was conducted by a student.
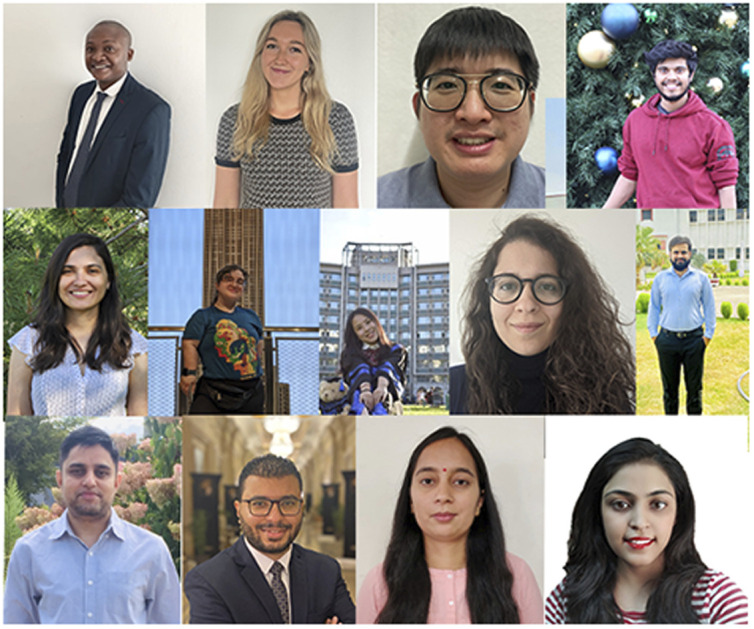

In 2021, *RSC Advances* launched an award series to recognise the hard work of students. These awards recognise outstanding work published in the journal, for which a substantial component of the research was conducted by a student. In order to be eligible for this award, the article must have been a research article and the first author or co-first author must have been a student at the time of carrying out the research. I’m delighted to say that we received over 900 nominations, which were shortlisted based on a number of criteria, and the winning papers were then selected by our Editorial Board and Associate Editors. Below, we highlight the winner of each subject category, and highlight the research that they carried out that led to them being chosen as a winner of this award.

***Analytical chemistry*** 
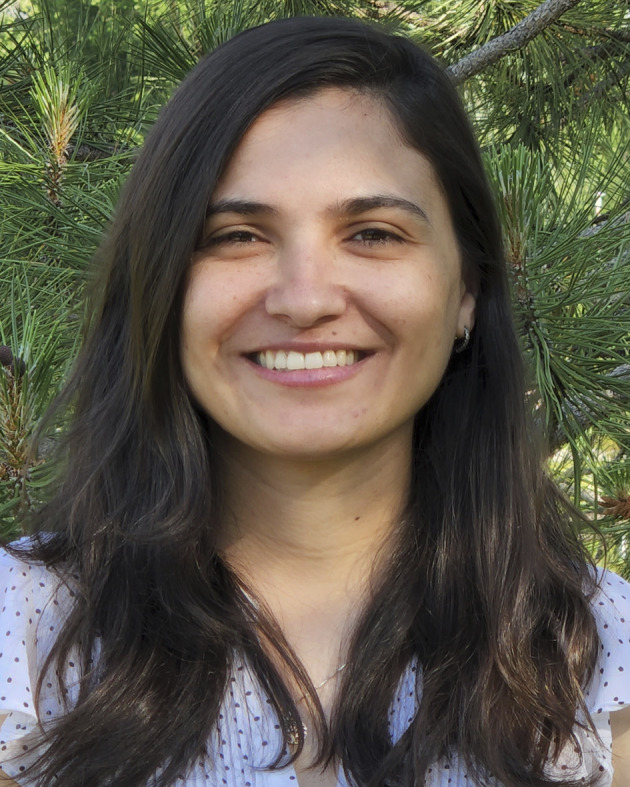


Vanessa N. Ataide, São Paulo University, Brazil

Vanessa is recognised for her outstanding contribution in the research advance presented in *Enhanced performance of pencil-drawn paper-based electrodes by laser-scribing treatment* (https://doi.org/10.1039/D0RA08874A).

Vanessa graduated in Chemistry (2015) from Presbyterian Mackenzie University. She received her MSc (2018) from the Institute of Chemistry of the University of São Paulo under the supervision of Prof. Dr Thiago R. L. C. Paixão. She is currently a PhD student with the same supervisor and in the same institution. Her research interests include electrochemical paper-based devices, carbon materials, fabrication of electrochemical sensors using low-cost techniques, and analytical applications involving clinical and environmental interest species. She receives financial support from São Paulo Research Foundation – FAPESP (Grant Number: 2018/14462-0). She is currently doing an internship at Colorado State University under the supervision of Prof. Dr Charles S. Henry, supported by FAPESP (Grant Number: 2021/10388-2). She is developing carbon-based low-cost electrochemical devices for the detection of Covid-19.

***Biological & medicinal chemistry*** 
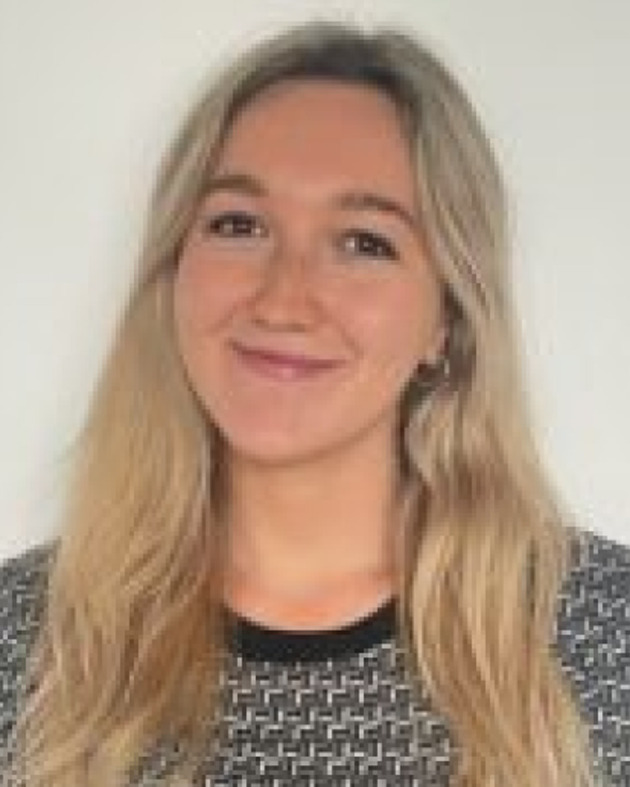


Nova O. Dora, University of Kent, UK

Nova is recognised for her outstanding contribution in the research advance presented in *Supramolecular self-associating amphiphiles (SSAs) as nanoscale enhancers of cisplatin anticancer activity* (https://doi.org/10.1039/D1RA02281D).

Nova grew up in London and developed a strong interest in science whilst at school. She went on to complete her undergraduate degree in biology at the University of Kent, Canterbury, during which she completed her final year research project investigating mechanisms of drug resistance in cancer cell lines. This was an area of great interest and so Nova then stayed at the University of Kent to complete a MScR investigating the potential of supramolecular self-associating amphiphiles as novel cancer treatments. After completing her masters program, Nova completed a PGCE in secondary education and is now a science teacher at a secondary school in West London. In her free time Nova likes to partake in sports such as netball and swimming and enjoys travelling and reading.

***Catalysis*** 
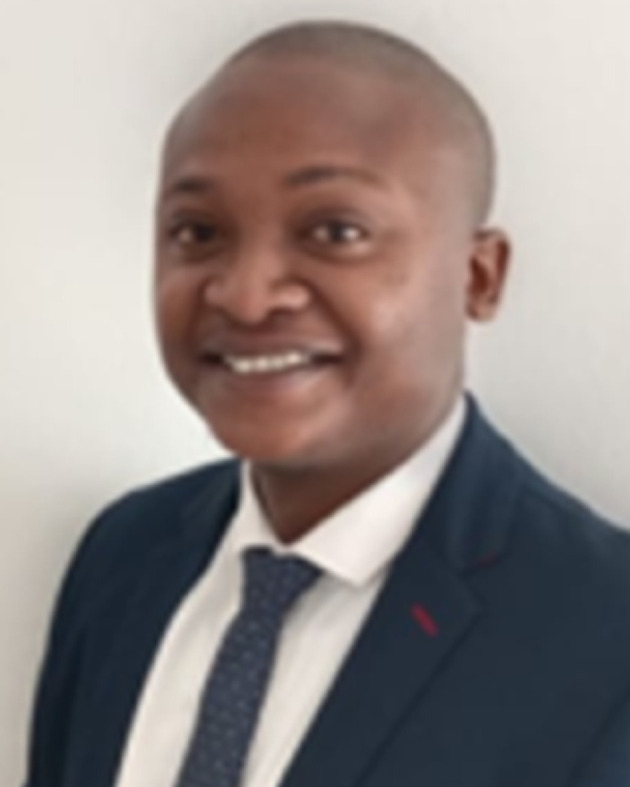


Jairus L. Lamola, University of Johannesburg, South Africa

Jairus is recognised for his outstanding contribution in the research advance presented in *Evaluation of P-bridged biaryl phosphine ligands in palladium-catalysed Suzuki–Miyaura cross-coupling reactions* (https://doi.org/10.1039/D1RA04947J).

Jairus Lamola was born in Sebokeng, a small township in Gauteng Province, South Africa. He graduated with BSc chemistry and biochemistry, and BSc(Hons) chemistry degrees from the University of Johannesburg (South Africa) in 2015 and 2016, respectively. He then obtained a Master’s degree in organic chemistry, in 2018 under the supervision of Dr Edwin Mmutlane. He received the Faculty of Science Dean’s award for the best final-year BSc student in 2015 as well as the top third-year student awards in chemistry and biochemistry (2015).

He started his PhD studies in organic chemistry in 2019 under the supervision of Prof. Chris Maumela and co-supervision of Prof. Cedric Holzapfel and Dr Paseka Moshapo. His doctoral research focuses on the design and development of novel P-bridged biaryl phosphine ligands for palladium-catalysed cross-coupling reactions. The PhD study has so far resulted in the publication of four research articles in international peer reviewed journals. Outside of work, he also enjoys cooking, storytelling, admiring nature and its biodiversity.

***Computational and theoretical chemistry*** 
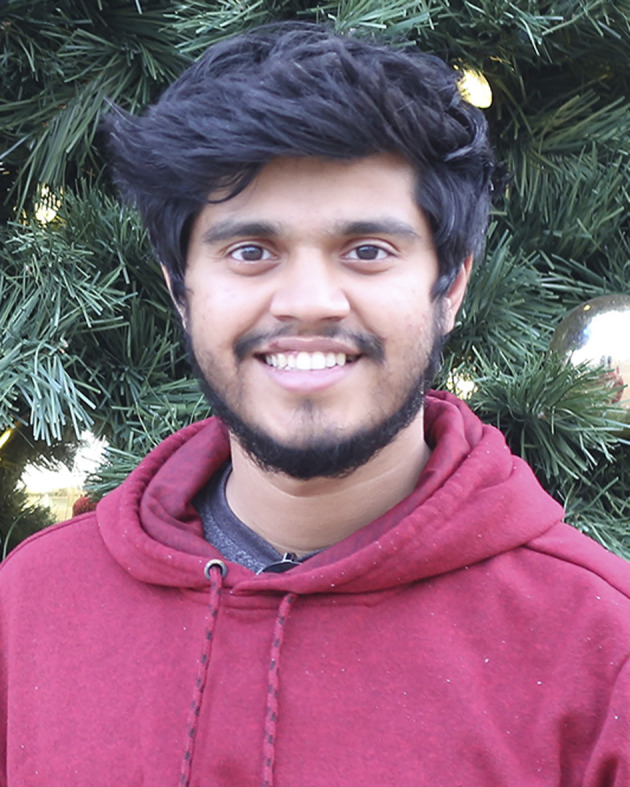


Abhishek T. Sose, Virginia Tech, USA

Abhishek is recognised for his outstanding contribution in the research advance presented in *Modelling drug adsorption in metal–organic frameworks: the role of solvent* (https://doi.org/10.1039/D1RA01746B).

Abhishek Tejrao Sose is currently a fourth-year PhD student working under the supervision of Dr Sanket Deshmukh in the Department of Chemical Engineering at Virginia Tech. His research is focused on the integration of the newly emerging field of artificial intelligence (AI) with molecular dynamics (MD) and Monte Carlo (MC) simulations to accelerate the design of new hybrid materials including metal–organic frameworks (MOFs), for biomedical and energy applications. A large part of his research also involves the development of accurate and transferable all-atom (AA) and coarse-grained (CG) models that are accelerated by optimization algorithms.

After finishing his bachelor’s degree in chemical engineering at the Indian Institute of Technology (IIT) Bombay in 2017, Abhishek decided to pursue his doctoral studies at Virginia Tech. Thus far, he has published four peer-reviewed journal articles (including three first-authored articles) and given 6 oral presentations and 5 poster presentations, at national and international conferences. Recently he was awarded the ‘Best poster award’ at the Macromolecules Innovation Institute (MII) Technical Conference & Review 2022 at Virginia Tech, for his work on investigating the molecular-level interactions between polymers and functionalized metal–organic frameworks. Moreover, his work on the development of forcefield interactions between MoS_2_ and water was featured as a supplementary cover for the *Journal of Physical Chemistry C* (JPCC). His latest work on the ‘Investigation of structure and dynamics of water confined between hybrid layered materials of graphene, boron nitride, and molybdenum disulfide’ was published in the *Journal of Materials Science* as an invited article.

***Energy chemistry*** 
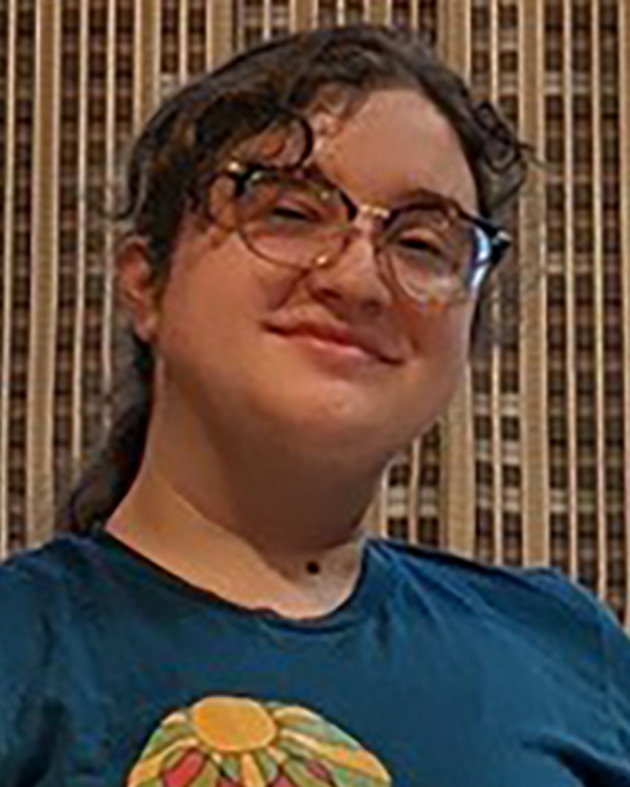


Alexandra H. Teodor, University of Tennessee at Knoxville and Oak Ridge National Laboratory, USA

Alexandra is recognised for her outstanding contribution in the research advance presented in *Aqueous-soluble bipyridine cobalt(**ii**/**iii**) complexes act as direct redox mediators in photosystem I-based biophotovoltaic devices* (https://doi.org/10.1039/D0RA10221K).

Alexandra Heather Teodor was born in 1995. She received her BS in biochemistry in 2016 from Virginia Polytechnic Institute and State University, focusing her studies on analytical and physical biochemistry. Alexandra then enrolled in the doctoral program of the joint University of Tennessee, Knoxville and Oak Ridge National Laboratory Graduate School of Genome Science and Technology. She joined the laboratory of Dr Barry Bruce to pursue her doctoral research in bio-hybrid electronic devices, furthering her interests in spectroscopy, physical, and electrochemical sciences. Alexandra graduated with her PhD in 2022, and accepted a job offer as a Space Photovoltaics Scientist for The Aerospace Corporation in California. She hopes to continue doing impactful work that will give back to the community.

***Environmental chemistry*** 
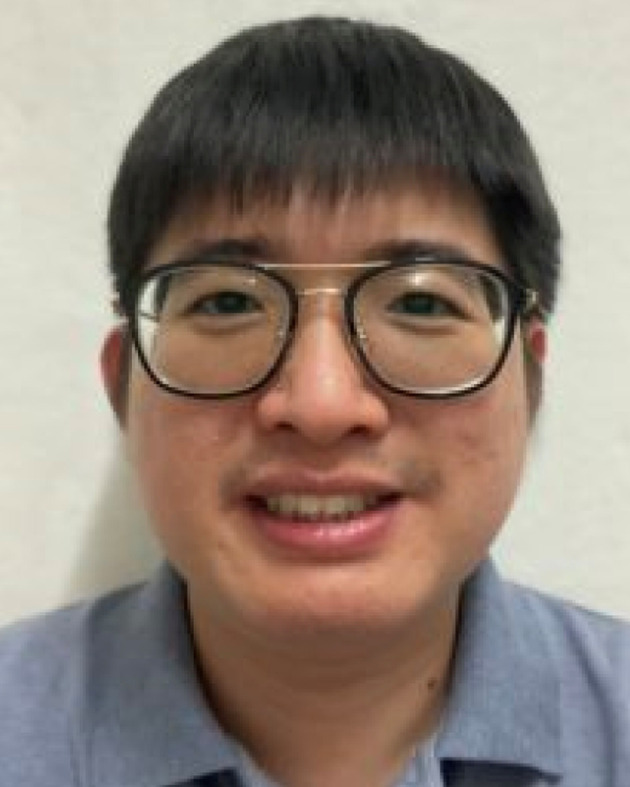


Yin Sim Ng, Universiti Sains Malaysia, Malaysia

Yin Sim Ng is recognised for his outstanding contribution in the research advance presented in *The enhancement of treatment capacity and the performance of phytoremediation system by fed batch and periodic harvesting* (https://doi.org/10.1039/D0RA08088H).

Yin Sim Ng was born and raised in Penang, Malaysia. He gained his BEng(Hons) in chemical engineering, from Universiti Sains Malaysia in 2014. During his undergraduate studies, he secured a JPA scholarship from the Public Service Department of Malaysia. He thereafter successfully registered himself as a Graduate Engineer with the Board of Engineers Malaysia (BEM). His passion and interest in biology and environmentally conscious design drove him to take up graduate studies in research related to phytoremediation and green technology (sustainable water and wastewater treatment). He joined Associate Professor Dr Derek Chan Juinn Chieh’s group specialising in biochemical processes and biotechnology involving plants, at the same faculty.

His Masters research focussed on phytoremediation studies in evaluating the exact phytoremediation rate (inorganics removal – ammonia, nitrate and phosphate) using an axenic method, and its performance in fish farm wastewater, aiming to enhance the treatment capacity and efficiency. He succeeded in isolation of the axenic cultures of *Hemianthus callitrichoides*, *Vesicularia montagnei* (Christmas moss), *Salvinia molesta*, *Spirodela polyrhiza*, and *Lemna* sp. for his study and side projects. He also received a travel bursary from the university to attend the International Phytotechnologies Conference in Hangzhou, China, that was organised by the International Phytotechnology Society (IPS), the Chinese Academy of Sciences (CAS) and the Institute of Soil Science, Chinese Academy of Sciences (ISSCAS) in Autumn 2016. He obtained MSc in chemical engineering in 2018. His doctorate studies concentrate on the role, mechanism, and mitigation of fouling from marine algae and their organics in membrane distillation systems. He obtained his MyMaster Scholarship from the Ministry of Education Malaysia and USM Fellowship for his studies. So far, he has published 8 international journal papers and 1 conference proceeding (ISI and Scopus indexed). He has been invited to perform 2 manuscript reviews for the *Journal of Hazardous Materials*. He is also a member of the Institution of Chemical Engineers (IChemE) and Microbiology Society, UK.

***Food chemistry*** 
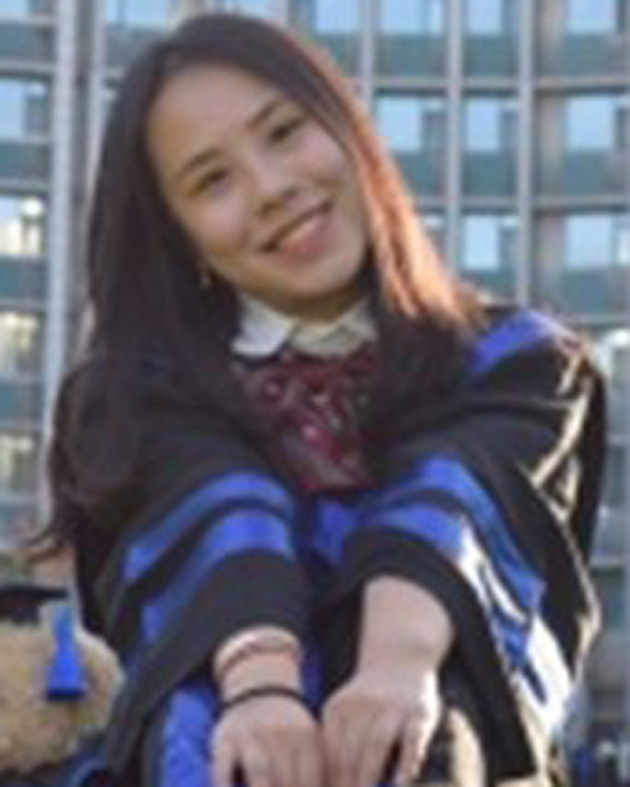


Yao Lu, Renmin University of China, China

Yao Lu is recognised for her outstanding contribution in the research advance presented in *Promotion effects of flavonoids on browning induced by enzymatic oxidation of tyrosinase: structure–activity relationship* (https://doi.org/10.1039/D1RA01369F).

Yao Lu received her MS degree from the Department of Chemistry, Renmin University of China in 2021, under the guidance of Prof. Rui-Min Han. Her research field is physical chemistry mainly concerning the interactions of flavonoids with tyrosinase. Her research interest is studying biochemical reaction mechanisms using optical spectroscopy.

***Inorganic chemistry*** 
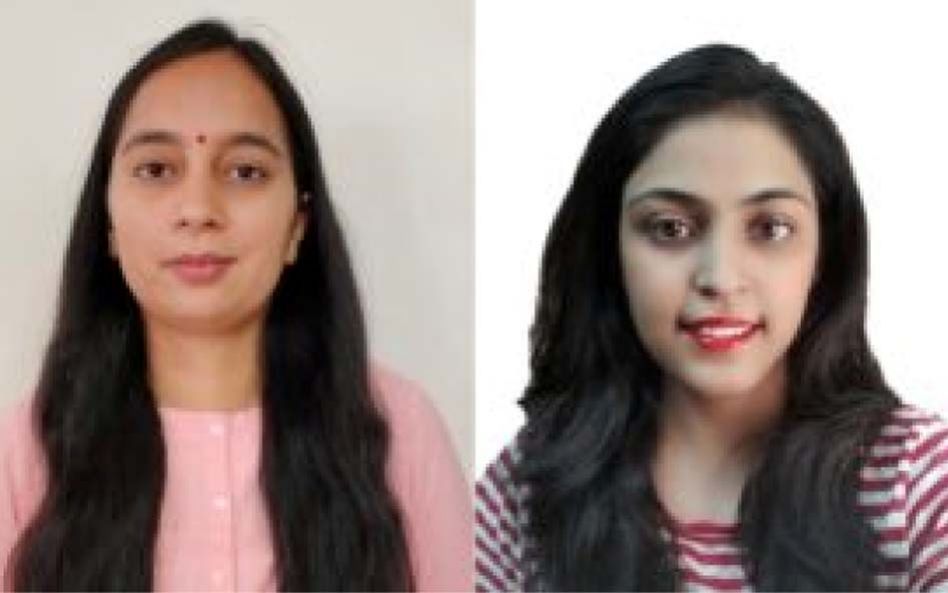


Aayushi Arora and Preeti Oswal, Doon University, India

Aayushi and Preeti are recognised for their outstanding contribution in the research advance presented in *Catalytically active nanosized Pd_9_Te_4_ (telluropalladinite) and PdTe (kotulskite) alloys: first precursor-architecture controlled synthesis using palladium complexes of organotellurium compounds as single source precursors* (https://doi.org/10.1039/D0RA08732G).

Ms Aayushi Arora who was born in Agra, India, in 1993, studied at Doon University Dehradun (2017-2021) for her PhD under the supervision of Dr Arun Kumar. She has been the recipient of the highly prestigious Indo-U.S. Fellowship for Women in STEMM (WISTEMM). With this fellowship, she carried out research work in 2020 at Texas A&M University, USA, under the supervision of Prof. John A. Gladysz. She has also been felicitated by the Hon’ble Governor of the State of Uttarakhand, India, at Rajbhawan on Uttarakhand Foundation Day, for her achievements as a young woman in science. Her research includes development and applications of new catalytic systems, designing fluorescent probes for sensing metal ions and Werner’s complexes for hydrogen bond donor catalysis. She has contributed to more than two dozen publications including articles and book chapters. In the short span of her career so far her *h*-index is 8.

Preeti Oswal was born in Himachal Pradesh, India, in 1995. After receiving her BSc and MSc degrees in chemistry in 2017, she became the recipient of a highly prestigious national DST-INSPIRE fellowship from the Department of Science and Technology (DST), Government of India, for pursuing PhD research for five years. For the last four and a half years she has been a PhD scholar at the Department of Chemistry, Doon University Dehradun, India, under the supervision of Dr Arun Kumar. She is working on designing novel organochalcogen and organophosphorous compounds which she uses as building blocks for catalysts and electrolysis. Her research experience includes homogeneous, heterogenous and nano-catalysis of various organic reactions such as Suzuki coupling, C–O coupling, aldehyde to amide transformation, allylation of aldehydes and Sonogashira coupling. She has also fabricated Pd_6_P at the nanoscale and explored its electrocatalytic application in hydrogen evolution reactions. At her young age and short span of her career so far, she has contributed to publishing more than 20 articles in journals of high repute, and 4 book chapters.

***Materials chemistry*** 
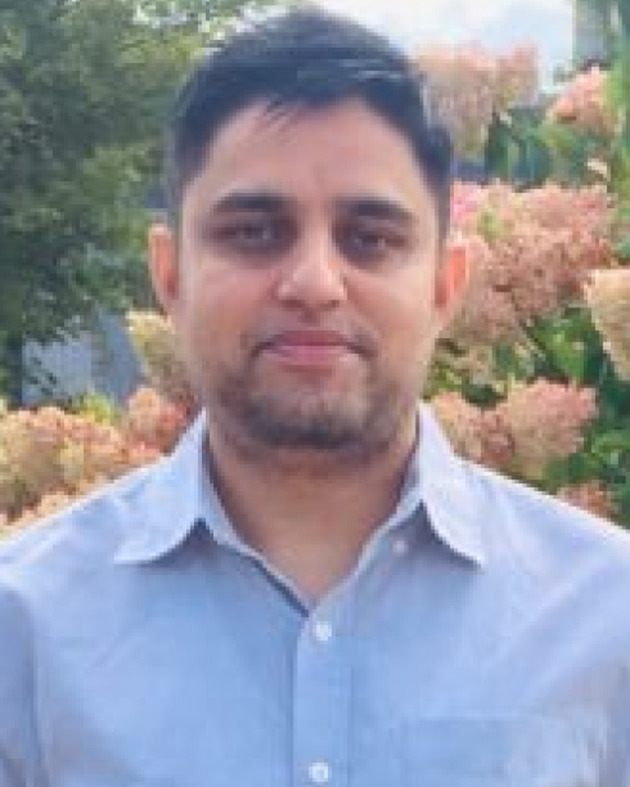


Shyam K. Pahari, University of Massachusetts, USA

Shyam is recognised for his outstanding contribution in the research advance presented in *Designing high energy density flow batteries by tuning active-material thermodynamics* (https://doi.org/10.1039/D0RA10913D).

Shyam Pahari is a doctoral candidate in inorganic chemistry at the University of Massachusetts Dartmouth, studying energy materials in the lab of Prof. Patrick Cappillino. His dissertation research focuses on designing high energy-density active materials for non-aqueous redox flow batteries by examining the effect of molecular structure on thermodynamic properties of electrolytes. In particular, he investigates the interplay between solvation-free energy and lattice enthalpy in determining active material solubility utilizing experimental and computational approaches.

Shyam is a first-generation college student and holds a MSci from Tribhuvan University, Kathmandu. Prior to joining UMass Dartmouth, he briefly worked as a high school chemistry teacher.

***Nanoscience*** 
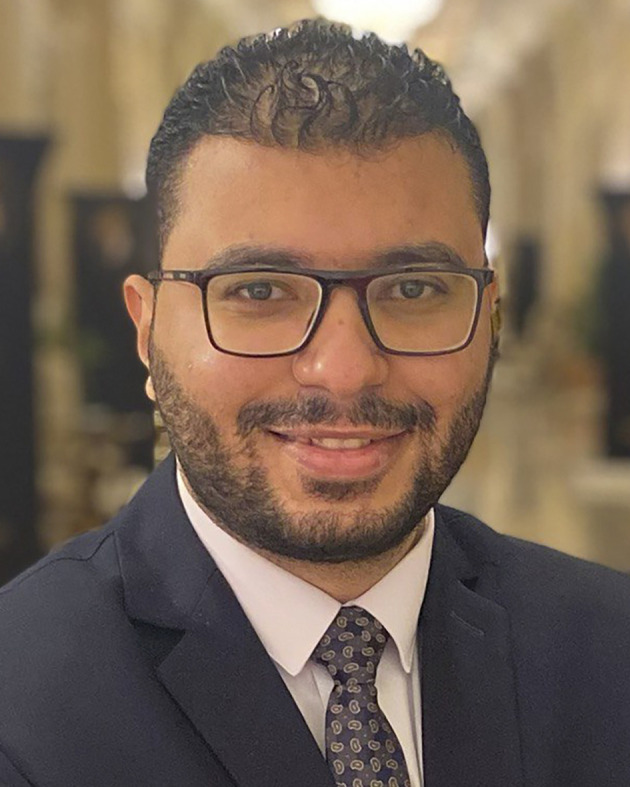


Mina Shawky Adly, Virginia Commonwealth University, USA

Mina is recognised for his outstanding contribution in the research advance presented in *Efficient removal of heavy metals from polluted water with high selectivity for Hg(ii) and Pb(ii) by a 2-imino-4-thiobiuret chemically modified MIL-125 metal–organic framework* (https://doi.org/10.1039/D1RA00927C).

Mina Shawky Adly is currently a lecturer of physical chemistry in the Chemistry Department, Faculty of Science, Mansoura University, Egypt. He earned his BSc at Mansoura University in 2012, and his Master’s degree in physical chemistry from the same university. He received a joint supervision grant from the Ministry of Higher Education from 2019 to 2020. He has worked under the supervision of professor Samy El-Shall at the College of Humanities and Sciences, Virginia Commonwealth University, USA. In his thesis, he pioneered metal–organic frameworks (MOFs) for different applications in adsorption and catalysis. He obtained his PhD in surface chemistry and catalysis in 2021 from Mansoura University. Recently, Mina’s research has focused on the synthesis of new MOFs and their applications related to the environment and energy, such as heavy metals removal, solar steam generation, and supercapacitors. He supervises research activities in the same field and teaches surface chemistry to Bachelor students in different programs at the Faculty of Science, as well as catalysis to students at the Faculty of Education. He has been involved in a collaborative research project financed by STDF in Egypt. He has seven publications in high impacted journals, including one in *JACS*.

***Organic chemistry*** 
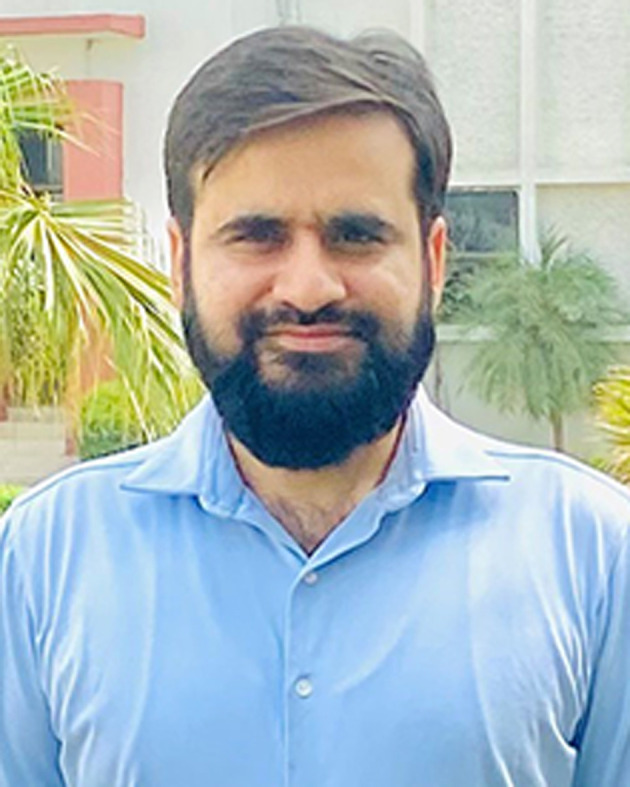


Ajaz Ahmed, Indian Institute of Integrative Medicine, India

Ajaz is recognised for his outstanding contribution in the research advance presented in *Conversion of *N*-acyl amidines to amidoximes: a convenient synthetic approach to molnupiravir (EIDD-2801) from ribose* (https://doi.org/10.1039/D1RA06912H).

Ajaz Ahmed was born and brought up in the Poonch District of Jammu and Kashmir. He received his BSc from Govt. Gandhi Memorial Science College Jammu, India, and MSc in organic chemistry from Bundelkhand University Jhansi, U.P., India. Following this, he joined the Indian Institute of Integrative Medicine (IIIM) Jammu, a laboratory under Council of Scientific and Industrial Research (CSIR), Jammu, Govt. of India in August 2017 as a junior research fellow after qualifying through the National Eligibility Test (NET) conducted by CSIR. He has passed various national level exams – CSIR-NET-JRF in 2016, CSIR-NET-JRF in 2017, GATE in 2016, GATE in 2019, and GATE in 2020, conducted by IIT. He has recently submitted his PhD thesis entitled “*N*-Glycosylation as a Tool Box for the Generation of Medicinally Important Nucleosides and Disaccharide Mimetics” to the Academy of Scientific and Innovative Research (AcSIR), under the supervision of Dr Debaraj Mukherjee, a principal scientist in the Natural Product and Medicinal Chemistry Division. His area of research is glycoscience which includes nucleoside chemistry, oligosaccharide synthesis, total synthesis of biologically active compounds, development of novel methods for glycosylation. and affordable routes for active pharmaceutical ingredients (API). He has 11 published papers in different reputed journals of organic chemistry and 2 review articles to his credit, and has also filed three patents related to API synthesis.

***Physical chemistry*** 
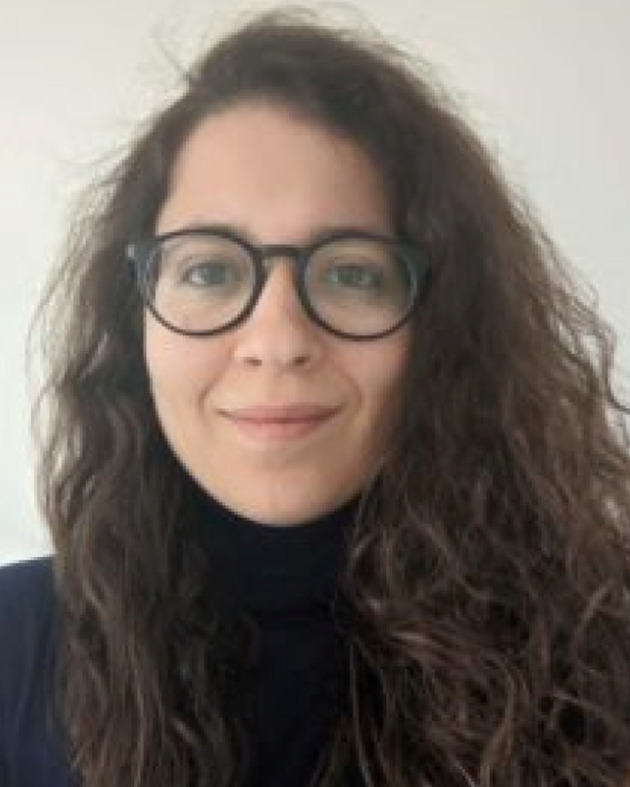


Rosaria Cercola, University of York, UK

Rosaria is recognised for her outstanding contribution in the research advance presented in *A “one pot” mass spectrometry technique for characterizing solution- and gas-phase photochemical reactions by electrospray mass spectrometry* (https://doi.org/10.1039/D1RA02581C).

Rosaria Cercola joined Caroline Dessent’s group at the University of York in 2015 as a PhD student, where she explored the gas-phase photochemistry of biological and pharmaceutical molecules.

She also developed a passion for science communication and outreach over her journey. She is now an editorial assistant at Science in School, the European journal for science teachers funded and supported by EIROforum.

Outside of work, Rosaria is the founder of “PhD and then what?” where she addresses themes like life abroad, the PhD journey and post-PhD careers.

Please welcome us in congratulating all of our winners!

We will continue to recognise any outstanding student contributions and plan to give out this award each year. If you published a manuscript in 2022, or go on to publish with the journal in the future, and recognise a significant contribution made by a student, please email advances-rsc@rsc.org to put forward the publication for consideration.

## Supplementary Material

